# The first detected airline introductions of yellow fever mosquitoes (*Aedes aegypti*) to Europe, at Schiphol International airport, the Netherlands

**DOI:** 10.1186/s13071-017-2555-0

**Published:** 2017-12-08

**Authors:** A. Ibañez-Justicia, A. Gloria-Soria, W. den Hartog, M. Dik, F. Jacobs, A. Stroo

**Affiliations:** 1 Centre for Monitoring of Vectors, Food and Consumer Product Safety Authority, P.O. Box 9102, 6700 HC Wageningen, The Netherlands; 2 Department of Ecology and Evolutionary Biology, Yale University, New Haven, CT 06511 USA

**Keywords:** *Aedes aegypti*, Airport introduction, Mosquito surveillance, Population genetics, Microsatellite markers

## Abstract

**Background:**

Air-borne introduction of exotic mosquitoes to Schiphol airport in the Netherlands has been considered plausible based upon findings of mosquitoes in aircraft cabins during 2008, 2010 and 2011. Beginning in 2013, surveillance efforts at Schiphol had focused on promptly detecting accidental introductions at the airport facilities in order to quickly react and avoid temporary proliferation or establishment of mosquito populations, identify the origin of the introductions, and avoid potential transmission of vector-borne diseases.

**Methods:**

BG-Mosquitaire mosquito traps were set at the most likely locations for arrival of the invasive *Aedes* mosquitoes as part of the mosquito monitoring program at Schiphol airport. Samples were collected bi-weekly. Upon detection of exotic specimens, information about the origin of the flights arriving to the particular location at the airport where specimens were captured was requested from airport authorities. The GIS tool Intersect was then used to identify airports of origin common to positive trapping locations during the specific trapping period. Captured *Aedes aegypti* mosquitoes were subsequently genotyped at 12 highly polymorphic microsatellite markers and compared to a reference database of 79 populations around the world to further narrow down their location of origin.

**Results:**

In 2016, six adult yellow fever mosquitoes were captured indoors and outdoors at the airport of Schiphol in the Netherlands confirming, for the first time, air-borne transport of this mosquito vector species into Europe. Mosquitoes were captured during three time periods: June, September and October. Containers carried by aircrafts are considered the most likely pathway for this introduction. GIS analysis and genetic assignment tests on these mosquitoes point to North America or the Middle East as possible origins, but the small sample size prevents us from reliably identifying the geographic origin of this introduction.

**Conclusions:**

The arrival of *Ae. aegypti* mosquitoes to Schiphol airport from flights arriving from overseas, demonstrates the potential risk of international flights to public health as carriers of arthropod vectors of disease. The results strongly suggest that disinsection of containers and their storage compartments inside the aircrafts could contribute to preventing future introductions of mosquito vectors. Invasive mosquito species introduced by aircrafts from overseas could become seasonally established during the warmer months in Europe, or permanently in certain climatically suitable areas for the species, with major consequences for human health.

**Electronic supplementary material:**

The online version of this article (10.1186/s13071-017-2555-0) contains supplementary material, which is available to authorized users.

## Background

Global spread of the yellow fever mosquito, *Aedes aegypti*, has historically been related to human trade and transport. In this way, the species has successfully colonized suitable locations in countries of all continents, except Antarctica. The northernmost documented occurrences in Europe (i.e. Bordeaux and Saint Nazaire, France; Swansea and Southampton, UK) arose from introductions *via* ships, and no evidence was found on the spread or establishment of the species at these places [1]. Historically, the species also displayed a much larger distribution in Europe compared to its current one [1].

In the nineteenth century, *Ae. aegypti*-driven outbreaks of yellow fever took place in port cities in northerly areas of Europe (e.g. Saint-Nazaire, France; and Swansea, UK). The twentieth century also witnessed one of the largest outbreaks of dengue recorded globally in Athens and neighboring areas of Greece in 1927–1928 [1]. Urban yellow fever was eliminated from many countries after the Second World War, by energetic campaigns to eliminate *Ae. aegypti* breeding sites through application of the pesticide dichlorodiphenyltrichloroethane (DDT) to infested containers and their surroundings [2].

In more recent times, globalization of trade and travel has facilitated the geographical spread of vectors and vector-borne diseases [3, 4], with air travel playing a major role in long-distance dispersal [5, 6]. In areas of high mosquito densities, mosquitoes can follow their human hosts unnoticed and enter aircrafts at the airports [7]. These aircrafts can then transfer mosquitoes from one location to another relatively rapidly, thus increasing their chance of mosquitoes surviving the trip and reaching the destination. Upon arrival in another country, mosquitoes may colonize new areas or infect people locally [7, 8]. For example, a mosquito which alighted from Bali with a military aircraft was considered the most likely source of a case of “airport dengue” in Australia in 2010 [9].

In 2008, the Netherlands reported live mosquitoes on a flight from Dar es Salaam, Tanzania, to Schiphol airport in Amsterdam, with several passengers complaining of being bitten on board. Mosquitoes collected by the flight attendants were identified as *Culex quinquefasciatus* Say [10]. A follow-up study in 2010 and 2011 focusing on answering basic questions as to the mosquito species, frequency, and number of mosquitoes on board of aircrafts that land at Schiphol airport, showed that exotic mosquito vectors of diseases (*Cx. quinquefasciatus*, *Culex antennatus* and *Aedes mcintoshi*) were transported on 10 of the 38 aircrafts inspected [11]. Air-borne movement of mosquitoes has thus proven to be possible based upon these findings. Given the high volume of air transport movements per year between Schiphol and vector-borne disease endemic regions in the world, establishment of mosquito vectors after introduction *via* aircraft should be considered a potential health risk.

Since 2013, surveillance efforts at Schiphol have focused on promptly detecting accidental introductions at the airport facilities, with the goal of eliciting a quick reaction to avoid temporary proliferation, identifying the origin of the introductions, and avoiding transmission of vector-borne diseases. Here we describe the methodology used for surveillance of mosquitoes at the airport of Schiphol and report the findings of yellow fever mosquitoes (*Ae. aegypti*) at the airport facilities in 2016. We also detail the vector-control response that followed these events. Efforts to identify the origin of the introductions combined data on flight departure locations provided by the airport authorities, the use of suitability maps, and genetic assignment tests of the specimens captured to a reference database of world populations based on 12 highly polymorphic microsatellite markers [12]. Identifying the origin of introduced invasive mosquito species is important to aid public health efforts to prevent the introduction and spread of these vectors of human-diseases.

## Methods

### Mosquito collection and identification

Since 2013, mosquito monitoring at Schiphol airport has mainly consisted of placing mosquito traps at the most likely locations for the arrival of invasive *Aedes* mosquitoes. Examples of such locations are: platforms of arrival gates with high volumes of international flights landings from overseas, platforms of gates for European flights arriving from areas where invasive mosquitoes are present (e.g. Italy, southern France), indoor locations where suitcases are unloaded, temporary storage locations for arriving cargo, imported fruits and vegetables, or animals.

The traps used at Schiphol airport are BG Mosquitaire traps (Biogents AG, Regensburg, Germany). These traps have been specifically developed for capturing *Aedes* mosquitoes (*Ae. albopictus*, *Ae. aegypti* and related species) and use a patented mix of artificial skin emanations (BG-Sweetscent), in combination with air convection and light-and-dark contrasts. Carbon dioxide is not used at the airport. Lure used in the traps has proven to be efficient for capturing adult *Ae. albopictus* and *Ae. aegypti* (both males and females) in the field [13–15]. Traps were continuously operated (24/7) and placed indoors and outdoors in shaded, wind-protected moist areas.

Mosquito samples were collected bi-weekly. Trapping nets from the BG trap with collected mosquitoes and a data form were sent together inside a sealed plastic bag to the laboratory of the National Reference Centre from the Food and Consumer Product Safety Authority (NVWA/NRC) for morphological identification. All data from each sampling location were submitted into VecBase (database of Centre for Monitoring of Vectors - CMV). In the laboratory, mosquitoes were counted and morphologically identified by specialists using the keys of Schaffner et al. [16] and Becker et al. [17]. Exotic *Aedes* species diagnosis was validated *via* a real-time PCR assay within the NVWA/NRC, specially developed for the CMV surveillance for the detection of *Ae. albopictus*, *Ae. aegypti*, *Ae. atropalpus* and *Ae. japonicus* [18]. In 2016, a maximum of ten mosquito traps were continuously functioning at the airport and routinely checked every two weeks. The action plan in place to respond to an invasive mosquito species introduction (IMS) is as follows: (i) Both the number of traps and their monitoring frequency are increased 2-fold at the airport to check for additional locations hosting these mosquitoes. (ii) Oviposition traps, black plastic containers filled with hay infusion and a piece of Styrofoam serving as the oviposition substrate, are deployed in the surroundings of the first findings to detect a possible establishment of the introduced species. At the airport, the top of the oviposition traps is covered with stainless steel bird netting to prevent the Styrofoam from been blown away by the aircraft engines. (iii) The search for IMS larvae is performed in potential breeding sites at the airport, prior to implementation of mosquito control interventions. Upon detection of *Ae. aegypti* in 2016, this plan was implemented as described.

### Flight origin data

Information about the origin of the flights arriving at each particular location (or gate) during the trapping period when exotic specimens were captured was requested from the airport authorities. A file was provided with the following information: date, flight number, estimated arrival time, origin (IATA Code), arrival platform and luggage handling area. The file was compared with the database Openflights.org [19] to find the name of the airport of origin, the city name, country, and geographical coordinates. Coordinates of the selected airports were input into ArcGIS 10.3 to generate a map indicating the candidate airports for bringing the exotic specimens during each positive trapping period. Using the intersect tool of ArcGIS we identified the airport origins common to several trapping location inputs during the desired period (that is, they intersect). Combining this map with suitability maps for *Ae. aegypti* mosquitos based on [20], we generated a new map identifying the airports around the world situated in areas were the target species could be present. Airports were considered as “non-suspected” of introducing the species when the species was absent and/or the probability of a mosquito population in the area was considered low.

### DNA extraction and genotyping

Total genomic DNA from *Ae. aegypti* adult legs was extracted using either High Pure PCR Template Preparation Kit (Roche, Basel, Switzerland), or the DNeasy Blood and Tissue kit (Qiagen, Venlo, the Netherlands), according to the manufacturer’s instructions, with an additional RNAse A (Qiagen) step. Samples were stored at -20 °C until further analysis. Individual mosquito material was genotyped as described in [21]. The microsatellite loci analyzed were: A1, B2, B3, A9 (tri-nucleotide repeats) and AC2, CT2, AG2, AC4, AC1, AC5, AG1 and AG4 (di-nucleotide repeats) [22]. Polymerase chain reactions were conducted as 10 μl reactions using the Type-it Microsatellite PCR Master Mix (Qiagen), 25 nM of each forward primer, 250 nM of each reverse primer, and 500 nM of a fluorescently labeled M13 primer. Thermocycler conditions were: 94 °C for 10 min, 35× (94 °C for 30 s, 54 °C for 30 s, 72 °C for 30 s), and 72 °C for 5 min. The resulting products were processed for fragment analysis at the DNA Analysis Facility at Science Hill at Yale University, USA, using GS 500 Rox internal size standard (Applied Biosystems, Foster City, USA). Microsatellite alleles were scored using GeneMapper v4.0 (Applied Biosystems). Raw allele frequencies of the reference panel are available at VectorBase.org. Raw microsatellite allele calls for the mosquitoes captured at Schiphol can be found in Additional file 1: Table S1.

### Population genetic analysis

Genetic assignment tests were performed in GENECLASS2 [23], using the Bayesian criteria for likelihood estimation with a threshold value of 0.05 [24]. The reference population panel included the 79 populations (*n* = 3632 individuals) from the six continents around the world where *Ae. aegypti* is present [12], and includes members from the subspecies *Ae. aegypti aegypti* as well as *Ae. aegypti formosus*. To estimate the accuracy of this individual assignment method using our reference panel, we selected 100 random individuals from the same panel (with at least one representative of each population) and re-assigned them to the reference panel without replacement (as if they were newly acquired samples). During this exercise, 76.2% of the individuals were correctly assigned back to their population of origin, and for 93% of the samples the correct population of origin was ranked among the top 5 assignment scores. It is worth noting that the individuals that were not accurately assigned to their original population were assigned to a population with a similar genetic signature and in close geographic proximity (e.g. Conch Key, FL rather than Miami, FL, USA). In addition to the individual assignment tests, the Netherlands’ individuals collected in the same period at the airport were also treated as belonging to the same population and a group genetic assignment was performed following the same criteria.

## Results

### Mosquito collection

The BG traps monitored every two weeks at the airport, only captured indigenous mosquitoes (at low abundance) in 2016 until June. These included: *Anopheles maculipennis* (*sensu* *lato*) (*n* = 17), *Culiseta annulata* (*n* = 15), and *Culex pipiens/torrentium* (*n* = 1316). Three adult *Ae. aegypti* were captured at two airport locations in June 2016 (see Table 1). One specimen was captured in a trap situated outdoors (Platform E), next to the arrival platform of intercontinental flights, and the other two specimens were captured indoors next to a mechanical unloading system named MUM [25], an innovative mechanical system that automatically unloads baggage/luggage containers, the type of containers used by airlines to transport suitcases. All three specimens were males, and all three were identified as *Ae. aegypti* using real-time PCR [18]. As a result of increasing the number of traps and trapping locations upon the first finding, one female *Ae. aegypti* was identified on September 13th, captured outdoors in a BG-trap at a different gate (Platform G), and on September 29th a second adult *Ae. aegypti* female was captured at a BG-trap at the MUM. The third and last finding (one female) was recorded in an indoor BG-trap placed in a baggage handling area of the airport (between platforms F and G) at the end of October (Table 1). The finding at this location was a consequence of the intensive surveillance implemented as part of the vector-control response. In total, six *Ae. aegypti* specimens were intercepted in 2016, both indoors and outdoors. These events can be considered as a minimum of a single introduction that persisted over the months, or as many introductions as the number of individual mosquitoes detected (*n* = 6). However, based on the distribution of the trapping periods and because none of the 27 infusion-baited oviposition traps placed in the surroundings of the first finding in June (Platform E, and MUM) were positive for exotic mosquitoes prior to and after the detection, we suspect three detected introduction events to Schiphol, in June (3 specimens), in September (2 specimens) and in October (1 specimen).

**Table 1:** *Aedes aegypti* findings in 2016 at Schiphol airport, the Netherlands

Date of placing^a^	Date of sampling^a^	Location	No. / Sex	Sample ID
24-05-2016	09-06-2016	Platform E (outdoors)	1 / Male	Neth16_802
24-05-2016	09-06-2016	Mechanical unloading system (indoors)	1 / Male	Neth16_845
09-06-2016	15-06-2016	Mechanical unloading system (indoors)	1 / Male	Neth16_853
29-08-2016	12-09-2016	Platform G (outdoors)	1 / Female	Neth16_6637071
12-09-2016	26-09-2016	Mechanical unloading system (indoors)	1 / Female	Neth16_6813416-1-85
17-10-2016	31-10-2016	Baggage handling (indoors)	1 / Female	Neth16_4388663

^a^Dates are reported in a day-month-year format

Larval sampling for IMS in all potential breeding sites at the airport was put in place prior to the mosquito control response. A total of 16 samples were collected containing exclusively indigenous mosquitoes of the genus *Culex* [pupae (*n* = 40), larvae (*n* = 946)]. A population of autogenous *Cx. pipiens* biotype *molestus* breeds in stagnant water in the basements of Schiphol airport. Samples were taken at those breeding sites, but only this species was present.

### Tracing the origin of aircraft flights

During the trapping-period when *Ae. aegypti* specimens were captured, flights from 206 airports around the globe landed at Schiphol airport (Table 2).

**Table 2:** Number of airport origins identified arriving at the trapping locations

Date of placing^a^	Date of sampling^a^	Location	No. of airport origins identified arriving at the trapping locations
24-05-2016	09-06-2016	Platform E (outdoors)	65
24-05-2016	15-06-2016	Mechanical unloading system (indoors)	58
24-05-2016	15-06-2016	Intersect (June)	36
29-08-2016	12-09-2016	Platform G (outdoors)	79
12-09-2016	26-09-2016	Mechanical unloading system (indoors)	61
29-08-2016	26-09-2016	Intersect (September)	26
17-10-2016	31-10-2016	Baggage handling (indoors)	179
24-05-2016	31-10-2016	Intersect (June-September-October)	14
24-05-2016	31-10-2016	All origins merged	206

^a^Dates are reported in a day-month-year format

**Fig. 1:**
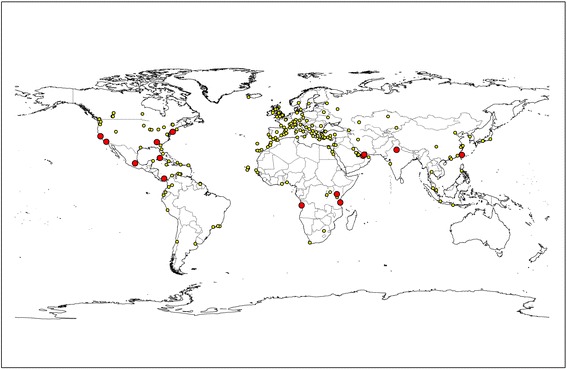
Origin of flights arriving at locations where *Ae. aegypti* has been found in Schiphol airport. All origins in June, September and October (*n* = 206; yellow dots). Intersect (common) origins June, September and October (*n* = 14; red dots)

Assuming that all *Ae. aegypti* specimens intercepted at the airport in June (indoors in the MUM, and outdoors) have the same origin, 36 possible airport origins were identified after combining the origin data from flights arriving at both sites during the catching period. Among others, flights from airports in North America, Middle East and Asia arrived to Schiphol during the findings in June at both locations (see Fig. 1).

Intersecting the data of airport origins from flights that arrived to the locations where the specimens were trapped in September (outdoors and indoors), we identified 26 potential origins that included airports in North America (including Florida), Middle East (Saudi Arabia) and Asia (see Fig. 1).

Data provided by the Schiphol authorities for the October findings provided 179 possible origins because this baggage location processes multiple arrivals. At this location, several baggage-handling companies work with a variety of container types or unload directly from the cargo space of the airliner to lorries in open air.

Assuming that all *Ae. aegypti* specimens intercepted at the airport originated from the same introduction, or that all specimens captured were carried in aircrafts proceeding from the same airport origin, we have identified 14 airports that originated flights arriving at all sites during the three catching periods. These airports are located in: Angola, Tanzania, Kenya, Panama, Mexico, Cuba, United States of America, United Arab Emirates, Taiwan and India (see Fig. 1).

### Population genetic analysis

The results from the genetic assignment analysis performed with GeneClass2 [23] on the *Ae. aegypti* specimens sent to Yale University to identify the likely origin of the introduction in Schiphol are shown in Table 3. Except for the last sample collected in October, all of the other five samples were successfully genotyped.

**Table 3:** Genetic assignment results from the *Aedes aegypti* specimens found at Schiphol airport, the Netherlands

Period^a^	Sample ID	Individual assignment^b^	Group A assignment^c^	Group B assignment^c^
24-05-2016 16-06-2016	Neth16_802	Saudi Arabia (87.350%)	Miami, FL, USA (99.95%); Rio, FL, USA (0.04%); VacaKeys,FL, USA (0.01%)	Saudi Arabia (79.85%); the Philippines (19.33%); New Orleans, USA (0.78%)
	Neth16_845	Miami (68.590%)		
	Neth16_853	Palm Beach (32.990%)		
29-08-2016 26-09-2016	Neth16_6637071	The Philippines (53.557%)	Saudi Arabia (95.47%); the Philippines (4.40%); New Orleans, USA (0.07%)	
	Neth16_6813416-1-85	Saudi Arabia (82.855%)		
17-10-2016 31-10-2016	Neth16_4388663	na	na	na

*Abbreviation: na* not available

^a^Dates are reported in a day-month-year format

^b^Individual frequency assignments; populations assigned the highest assignment score (0–100%)

^c^Group assignment test; populations assigned the highest assignment score (0–100%). The three locations with the highest assignment score are shown

When each individual was treated as an independent introduction, the analysis identified four potential sources, two from Asia (Saudi Arabia and the Philippines) and two from North America (Miami and Palm Beach in Florida, USA) (see Table 3). When individuals were grouped by the month in which they were captured and treated as two introductions: June (3 individuals) and September (2 individuals), the analysis identified two potential sources, Miami and Saudi Arabia with a 99.95% and 95.47% assignment probability, respectively (see Table 3). However, when the individuals are all considered part of the same population, either as a result of the introduction of multiple individuals at once, a population already established at the airport, or multiple introductions carried by different aircrafts originating at the same foreign airport, the highest assignment score indicates Saudi Arabia as the most likely source, followed by the Philippines (Table 3).

## Discussion

### Detection of first *Ae. aegypti* in Schiphol airport

The spread of *Ae. aegypti* around the world is most commonly associated with the transport of artificial containers infested by eggs or immatures [26]. However, the current study focuses on the movement of yet another developmental stage of great epidemiological importance: the adult mosquito. Schiphol airport in the Netherlands intercepted six adult yellow fever mosquitoes in 2016, confirming the possibility of air-borne introduction of this mosquito vector species to Europe, and the first finding of this species within a European airport facility. Aircraft arriving to Schiphol airport from areas hosting a large population of *Ae. aegypti* could introduce mosquitoes rapidly from abroad, thus increasing their chance of survival in receptive areas [7]. *Aedes aegypti* mosquitoes intercepted in June and in September at Schiphol airport were captured in outdoor and indoor traps. The trapping location choices, devices and lures used to detect this IMS (BG-mosquitaire with BG-Lure) proved to be effective for both male and female *Ae. aegypti*. Capture of male specimens is not unexpected, since *Ae. aegypti* males mate near their host [27]. The intercepted mosquitoes presumably traveled from North America or Asia, thus surviving a minimum of seven hours inside an aircraft during the flight. These mosquitoes then left the aircraft (or the containers) when doors opened and eventually flew to the traps. Finding of the specimens indoors, next to locations where sealed containers carrying personal baggage are opened for first time after arrival from other countries, suggests that *Ae. aegypti* could have either followed a cargo-handler inside the container and was trapped inside, or that the mosquito was attracted by the human odours emanating from the suitcases/baggage at the airport of origin. If the latter were true, cargo planes containing goods would probably be less attractive to this species than planes transporting humans and their personal belongings.

### Public health implications

Introductions of *Ae. aegypti* from endemic countries at this busy airport imply a major public health risk. The presence on board of mosquitoes from areas with endemic arthropod-transmitted diseases (e.g. malaria endemic areas) put passengers at immediate health risk through biting during the flight [28]. Upon arrival in another country, infected mosquitoes could transmit the pathogen locally in and around the airport, as is the case of “airport malaria” [7, 8]. *Aedes aegypti* transmits dengue virus, yellow fever virus, chikungunya virus and Zika virus, among other arboviruses [29–32]. Because of the short interval of time required for completing the journey, air transport has been associated with a higher risk of introducing a live infected mosquito compared to, for example, sea or road transport [9].

Suitability maps suggest that the climate in the Netherlands is not favorable for the survival and establishment of *Ae. aegypti* [20]. Unlike *Ae. albopictus* eggs, *Ae. aegypti* eggs cannot diapause, thus could not survive the outdoor Dutch winter conditions. Nevertheless, evidence suggests that *Ae. aegypti* could overwinter within human microhabitats in regions where the low temperatures would otherwise not allow them to survive [33]. Therefore, constant vector surveillance to prevent the introduction of invasive mosquito species beyond its native range is imperative to prevent transmission of pathogens transmitted by these mosquitoes.

### Vector control response

*Aedes aegypti* establishment strongly depends on the availability of artificial water-holding containers for oviposition and larval development. The presence of water-holding containers at Schiphol airport, prior to the *Ae. aegypti* findings, was considered low. However, as part of the vector-control response, operators of Schiphol facility services applied hot water pressure cleaning machines to the drainage water system and rain gutters at all terminals in order to eliminate any larvae/pupae that could be present and avoid water stagnation. Water containers found (stagnant water) were sampled for mosquito immatures and later preventively treated or eliminated. Wells with stagnant water, holes, and open PVC cable tubes containing water were also closed with insulation foam or concrete. Paraffin oil was added to stagnant water locations inside the buildings at the underground level. These strategies allow us to rule out an established population of this mosquito in the airport.

### Origin of *Ae. aegypti* introduced to Schiphol

Genetic assignment tests allow us to broadly identify the region of origin of *Ae. aegypti* intercepted at Schiphol. Similar analysis was performed on *Ae. aegypti* mosquitoes imported into the Netherlands in 2010, successfully tracking the origin of these mosquitoes to a tire shipment from Miami, Florida, USA [34]. However, in this introduction at the airport, the specific location could not be further narrowed down due to the small number of specimens collected and the uncertainty of whether they belonged to the same source population or multiple sources. Furthermore, the ability of the genetic tests to identify the source of the introduction is limited by the representation of the source area in the genetic reference dataset. Nevertheless, populations of *Ae. aegypti* display a strong hierarchical geographic signature [11]. Thus, even if the source population was absent from the dataset, the genetic assignment test will point to a geographic population represented in the dataset that shares a genetic signature with the source location, a geographically close location. One should also bear in mind that a portion of containers arriving to the airport could have been transferred from other routes at one or multiple airports prior to the final arrival and information of prior transfers was not available from the airport authorities.

Since the mosquitoes were not trapped inside the aircrafts but at the airport facilities, one should consider the possibility that *Ae. aegypti* was already present on the ground. The mosquitoes captured could be remnants of the earlier introduction to the Netherlands in 2010 [34]. However, we consider this unlikely because the *Ae. aegypti* individuals intercepted in 2010 were breeding in tire yards, that were treated with adulticides and larvicides at the time and the follow up on these vector control measures determined that the elimination was successful [35]. The tire yard and the airport are separated by more than 70 km and there has not been any report of *Ae. aegypti* in the time interval between the 2010 and 2016.

### Future recommendations

Identifying the origin of introduced invasive mosquito species is relevant to aid public health efforts to prevent the introduction and spread of disease vectors. In the case of Schiphol airport, upon knowing the exact origin of the flights transporting the species and the companies involved, the following measures could be put in place: (i) require the involved airline agencies to execute special disinsection measures at the airport of origin inside the containers; (ii) inform airport authorities so they can handle the containers from infested origins in quarantine areas designed to prevent possible introductions; (iii) inform the European invasive mosquito surveillance teams about the findings, especially from European regions with a climate suitable for the establishment of invasive mosquitoes in Europe (Turkey, Spain, Italy, etc.).

The current recommendations of the WHO for aircraft disinsection with insecticides [36], include specifications for aerosols and approved insecticidal formulations, and include three preferred methods: disinsection before take-off (“blocks-away” disinsection), disinsection top-of-descent (spraying of the cabin is done immediately after the aircraft started its descent to destination), and disinsection with residual insecticide. Disinsection on the ground (“on-arrival” disinsection) may be retained as an acceptable back-up method if an aircraft, coming from areas of threat, has not been adequately disinsected by any of the preferred methods [36]. The finding of specimens at the baggage handling area suggest that disinsection did not take place inside the transported containers, nor within the baggage compartment of the aircraft. Alternatively, it may have been applied and was not efficient, and therefore could lead to subsequent introductions of invasive mosquitoes. Based upon the evidence presented above, the authors consider that for a thorough disinsection, it is of crucial importance to include the containers and the container compartments of the aircrafts.

Additionally, sample degradation during trapping or handling of specimens, or a limited amount of biological material for genetic analysis, can compromise subsequent genetic work. Thus, if the genetic analysis were to be incorporated as part of the vector control program, it is necessary to establish protocols aimed at maximizing the information obtained from the specimens collected, and to promote close communication between the vector control agencies and the genetic test facilities. For example, the number of mosquito traps (lured and ovitraps) could be increased at the airport to increase the detection power and subsequently, obtain more information about the introductions and provide more material for the genetic analysis. It is also recommended to increase the sampling frequency to be performed weekly instead of fortnightly at the airport. Additionally, increasing the representation of candidate populations in the genetic reference panel would significantly improve the resolution of the genetic assignment method for future analysis.

## Conclusions

The presence of *Ae. aegypti* mosquitoes at Schiphol airport from flights arriving to the Netherlands, demonstrates the potential risk of international flights for public health. Identifying the origin of introduced invasive mosquito species is relevant to aid public health efforts to prevent the introduction and spread of these vectors of human-diseases. Collaborations between vector control agencies, airport authorities and genetic laboratories can facilitate tracking the origin of novel introductions. Furthermore, baggage compartments in aircrafts should be considered as potential pathways for the introduction of mosquitoes and, if disinsection is put in place, should also include these compartments in the aircraft.

## Additional file

Additional file 1: Table S1. Genotypes of the five *Aedes aegypti* specimens captured at Schiphol, the Netherlands (2016) at 12 microsatellite loci. Loci AC1, AC2, AC4, AC5, CT2, AG1, AG2, and AG5 are dinucleotide repeats; loci A1, A9, B2, B3 are trinucleotide repeats as described in Gloria-Soria et al. [12]. (XLSX 10 kb)
